# Perineural Spread of Orbital Immunoglobulin G4-Related Disease: A Case Report

**DOI:** 10.7759/cureus.77790

**Published:** 2025-01-21

**Authors:** Harshini Sirvisetty, Samuel Bockhorst, Ramin Hamidi

**Affiliations:** 1 Department of Radiology, University of Louisville School of Medicine, Louisville, USA; 2 Diagnostic Radiology, University of Louisville Hospital, Louisville, USA

**Keywords:** azathioprine, corticosteroids, granulomatosis with polyangiitis, immunoglobulin g4-related disease, lymphoma, orbital involvement, perineural spread, rituximab, sarcoidosis, thyroid ophthalmopathy

## Abstract

Immunoglobulin G4-related disease (IgG4-RD) is a rare, immune-mediated fibroinflammatory condition that can affect multiple organs. It is characterized by elevated serum IgG4 levels, lymphoplasmacytic infiltration, storiform fibrosis, and obliterative phlebitis. Diagnosis can be challenging due to limited awareness amongst clinicians and its wide range of clinical manifestations. We present a case of an elderly woman with bilateral orbital IgG4-RD and perineural spread, a rare manifestation of the disease. Serological testing showed markedly elevated IgG4 levels, and imaging revealed bilateral extraocular muscle enlargement and inflammatory involvement of the lacrimal glands, orbital fat, and trigeminal nerves. The patient initially responded to oral prednisone but discontinued therapy due to side effects. Rituximab, a second-line treatment, caused anaphylaxis, prompting a switch to azathioprine, which led to normalization of IgG4 levels and resolution of symptoms. This patient's positive response to azathioprine highlights the importance of individualized therapeutic strategies in managing this complex condition. This case also underscores the importance of recognizing IgG4-RD as a potential cause of orbital and neurological symptoms. Increased awareness of its diverse clinical and imaging features can facilitate early diagnosis and treatment. To date, this is one of the few documented cases of orbital IgG4-RD with perineural spread, emphasizing the need for further research and reporting to enhance understanding of this rare manifestation.

## Introduction

Immunoglobulin G4-related disease (IgG4-RD) is a rare yet rapidly evolving immune-mediated fibroinflammatory condition that can involve multiple organs. Initially, many manifestations of IgG4-RD were thought to be isolated single-organ diseases. However, in 2000, a relationship between the diverse clinical features and elevated serum IgG4 levels was established [1]. The most common presentations of IgG4-RD include autoimmune pancreatitis, sialadenitis, tubulointerstitial nephritis, and dacryoadenitis, amongst others. Histologically, affected tissues are characterized by lymphoplasmacytic infiltration, storiform fibrosis, and obliterative phlebitis [2]. Organs involved exhibit tumefactive enlargement, with ductal tissues displaying circumferential wall thickening [3]. Awareness and thorough understanding of IgG4-RD is imperative as the disease can mimic many malignant and inflammatory disorders, yet it responds to unique therapeutic interventions. In the context of periorbital tissues, differential diagnoses include lymphoma, Graves' orbitopathy, granulomatosis with polyangiitis, and sarcoidosis [4]. Due to the variability in clinical presentation, a definitive diagnosis necessitates a combination of serology, biopsy, and high index of suspicion. If the orbits or cavernous sinus are involved, delayed diagnosis and treatment can manifest in complications such as orbital pseudotumor, proptosis, vision loss, and/or cranial nerve palsies [5]. 

The epidemiology of IgG4-RD remains poorly understood due to its rarity and limited diagnostic awareness, likely leading to an underestimation of its true prevalence. It is known, however, that middle-aged to elderly males are disproportionately affected, with a male-to-female ratio of three to one [6]. This is an important distinction to make from similarly presenting autoimmune diseases, which are more predominant in females. Interestingly, IgG4-RD involving the head and neck, such as orbitopathy and sialadenitis, shows no gender predilection [4]. 

A few reports have explored the diverse manifestations of IgG4-RD in the head and neck, particularly hypertrophic pachymeningitis and hypophysitis. Here, we present one of the limited cases of perineural spread of orbital IgG4-RD. By highlighting this uncommon and variable presentation, we aim to enhance clinical recognition and mitigate delays in appropriate diagnosis and treatment. 

## Case presentation

A 63-year-old Black woman with a history of asthma, anxiety/depression, and hypertension presented with a two-year history of bilateral upper eyelid swelling and daily floaters in her left eye for the past year. She endorsed persistent eye itching, watering, a sensation of orbital pressure, and blurry vision. On examination, proptosis was greater in the left eye compared to the right, with bilateral upper lid myxedema and orbital fat prolapse. The left eye showed a restricted upward gaze accompanied by discomfort. Fundoscopic examination revealed an epiretinal membrane in the left eye and a choroid nevus measuring two disc diameters. Hertel exophthalmometry readings were 22|25, with no prior measurements available for comparison. 

**Figure 1:**
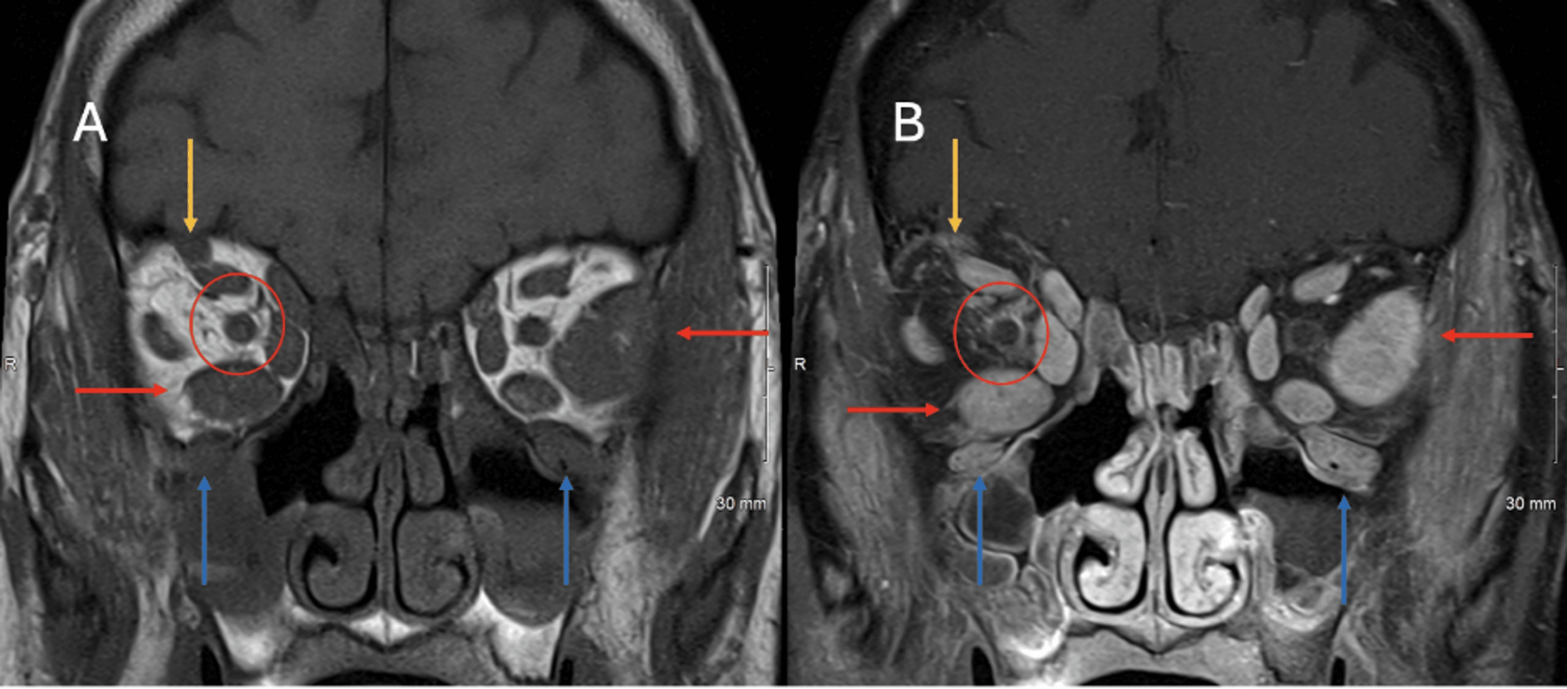
Coronal MRI scans of orbits with and without contrast Coronal T1 weighted MR image without contrast (A) and coronal fat-saturated T1 weighted MR image at the same level with contrast (B). There is thickening with enhancement of the supraorbital (orange arrow) and infraorbital (blue arrows) nerves. A red arrow (pointing to the left) and another red arrow (pointing to the right) demonstrate the inferior and lateral rectus muscles. There is an asymmetric enlargement of enhancing extraocular muscles, most notably the left lateral rectus. Peri-ophthalmitis about the right optic nerve sheath manifests as nerve sheath thickening with enhancement and adjacent fat stranding, identified to best advantage on the fat-saturated T1 post-contrast images. All findings are related to IgG4-RD. Incidental note made of post-operative changes of paranasal sinuses with inflammatory mucosal hypertrophy and mucosal retention cysts (likely not related to IgG4-RD). IgG4-RD - immunoglobulin G4-related disease

**Figure 2:**
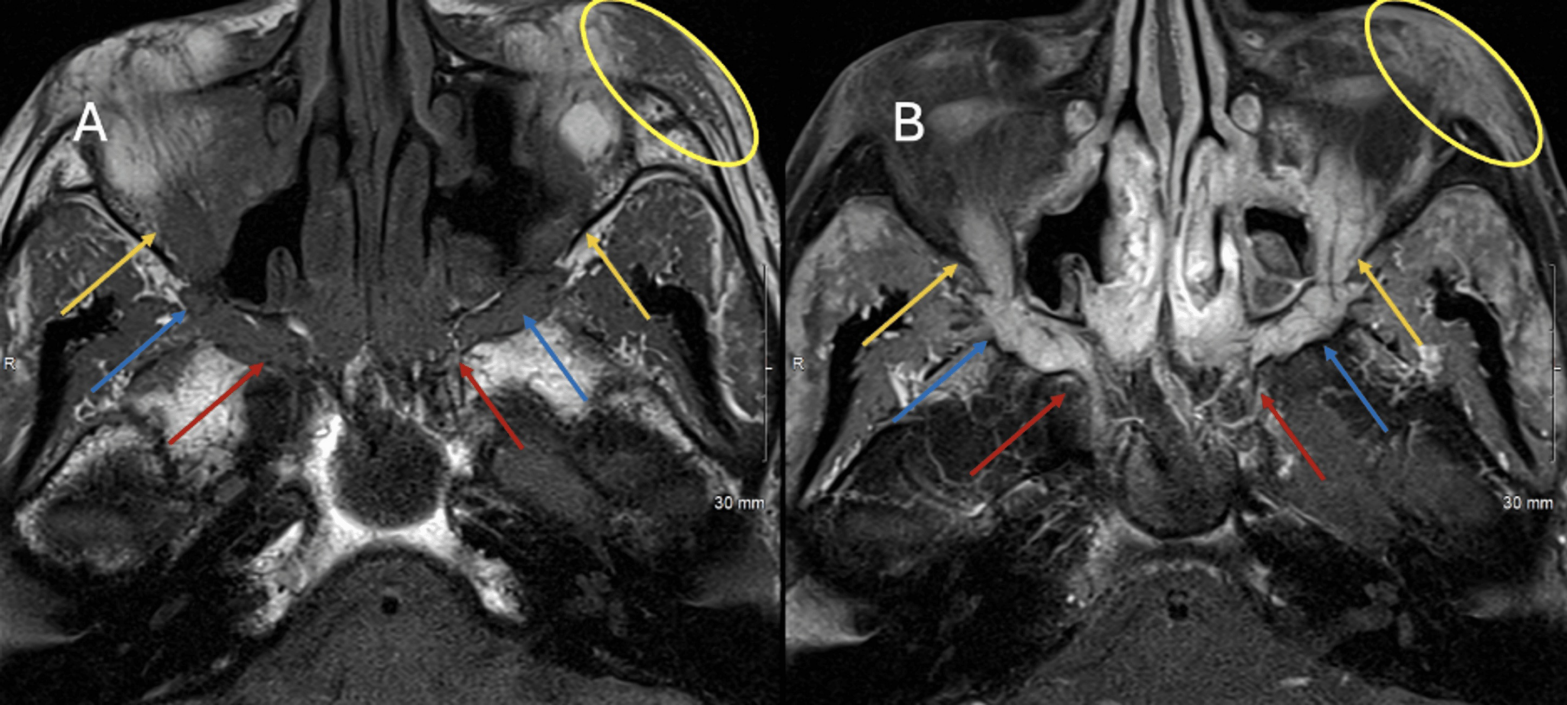
Axial MRI scans of orbits with and without contrast Axial T1 weighted MR image without contrast (A) and axial fat-saturated T1 weighted MR image at the same level with contrast (B). There is enlargement with thickening of the left lacrimal gland (yellow oval) related to IgG4-RD. Furthermore, there is thickening with an enhancement of the infraorbital nerves bilaterally within the infraorbital nerve canal (orange arrows), pterygopalatine fossa (blue arrow), and entering the foramen rotundum (red arrows). On the right side, infraorbital nerve enhancement extends through the foramen rotundum into the expected location of Meckel's cave. IgG4-RD - immunoglobulin G4-related disease

Coronal MRI scans of the orbits with and without contrast demonstrate bilateral enlargement of the extraocular muscles, orbital nerves, and peri-ophthalmitis (Figures 1A-B). Axial MRI scans of the orbits with and without contrast demonstrate infiltrative enlargement of the left lacrimal gland and perineural extension of disease along the infraorbital nerves (Figures 2A-B). The differential diagnosis includes IgG4-related disease, sarcoidosis, lymphoma, atypical thyroid ophthalmopathy, and granulomatosis with polyangiitis. 

The patient had improved swelling and symptoms with oral prednisone; however, she also had an intolerable rash. Laboratory evaluation demonstrated an exceptionally elevated level of IgG4 of 652.7 mg/dL (normal: 2.5 mg/dL - 135 mg/dL). Antineutrophil cytoplasmic antibody (ANCA) panel and antinuclear antibody (ANA) were negative, and both lysozyme and rheumatoid factor levels were within normal limits. Hematology/oncology was consulted, and after finding no other site of IgG4-RD, rituximab therapy was initiated. 

Despite proper premedication, the patient experienced anaphylaxis during her first rituximab infusion, developing acute shortness of breath 50 minutes into the treatment. Although she did not have chest pain, abdominal symptoms, or rash, her troponin levels increased from 24 to 166, which required hospitalization and was attributed to the infusion reaction and administered epinephrine. Due to this reaction, rituximab was discontinued, and the patient was transitioned to oral azathioprine therapy, which was continued as a home medication. 

After one month on azathioprine, the patient reported significant improvement, including reduced diplopia and swelling and no eye pain. At her three-month ophthalmology follow-up, motility was restored, and swelling had markedly improved. Per oncology, she will be maintained on azathioprine with quarterly blood work and plans for annual oncology follow-up. Most notably, her recent IgG4 subclass levels normalized to 14 mg/dL. She is scheduled for a six-month ophthalmology reassessment.

## Discussion

IgG4-RD 

The pathophysiology of IgG4-RD involves a complex interplay of innate and adaptive immunity, with CD4+ T-cell polarization promoting fibrosis through innate immune cells and B-cells generating IgG4-secreting plasmablasts while sustaining memory CD4-positive T-cells [4]. The synergy of these mechanisms underpins the fibroinflammatory pathology of IgG4-RD. Current evidence suggests that IgG4 itself is not the primary driver of the disease [4,7]. While elevated serum IgG4 is a hallmark, it is not a reliable standalone marker; an IgG4-to-total IgG ratio >40% is more indicative of disease [8]. Typical cases exhibit a ratio exceeding 70%, and ratios approximating 40% should be interpreted with caution [2]. Serum IgG4 concentrations exceeding 500 mg/dL are considered 90% specific for IgG4-RD [9], a threshold surpassed in our patient. At presentation, her serum IgG4 concentration was markedly elevated, although the IgG4-to-total IgG ratio could not be calculated due to missing data. Following treatment, her ratio decreased to 6%, reflecting a significant therapeutic response. 

Tissue biopsy is critical to a definitive diagnosis, requiring >50 IgG4-positive plasma cells per high-power field (hpf), averaged across three fields [10]. Additionally, the presence of two out of three characteristic histological features - lymphoplasmacytic infiltration, storiform fibrosis, and obliterative phlebitis - is required [2]. It should be noted, however, that IgG4-RD involving lacrimal glands often lacks storiform fibrosis and obliterative phlebitis [4]. Tissue eosinophilia may also be prominent, as observed in our patient, who had an eosinophil count of 722 (8.6%). Given the imaging findings and markedly elevated serum IgG4 levels (>500 mg/dL), a biopsy was deemed unnecessary in this case. 

Radiological features of IgG4-RD orbitopathy include symmetric enlargement of extraocular muscles, cranial nerves, and lacrimal glands. Mass lesions demonstrate homogeneous internal architecture, well-defined margins, and contrast enhancement [7]. CT and MRI findings, such as infraorbital nerve enlargement on MRI, can be highly suggestive of ocular IgG4-RD. 18F-fluorodeoxyglucose positron emission tomography (PET) can aid in assessing systemic disease involvement [11]. In our patient, MRI findings were consistent with IgG4-RD, showing infiltration of extraocular muscles, lacrimal glands, and trigeminal nerves. While imaging can strongly suggest IgG4-RD in straightforward clinical presentations, histopathological confirmation remains indispensable. 

IgG4-RD necessitates ongoing monitoring due to recurrence risk. Glucocorticoids are the first-line therapy; disease-modifying antirheumatic drugs (DMARDs), such as azathioprine or cyclophosphamide, are considered when glucocorticoid toxicity or contraindications arise [5]. Rituximab, a B-cell depleting agent, is an effective option for patients unlikely to achieve remission with glucocorticoids alone, particularly in cases with high IgG4 levels. Rituximab reduces IgG4-secreting plasmablasts, addressing the underlying pathology [4]. In this patient, glucocorticoid therapy was effective initially but discontinued due to intolerable side effects. Rituximab caused anaphylaxis, prompting a switch to azathioprine, which led to excellent clinical improvement and normalized serum IgG4 levels. Radiographic reevaluation is pending. 

Orbital IgG4-RD and perineural spread 

Orbital IgG4-RD commonly presents as dacryoadenitis and lacrimal gland involvement and also affects the extraocular muscles, with a predilection for the lateral rectus, sclera, uvea, and nasolacrimal duct [12]. Clinically, patients may exhibit proptosis, ptosis, restricted eye motility, and, in severe cases, optic neuropathy and blindness [13]. Our patient exhibited classic radiographic and clinical findings characteristic of IgG4-RD orbitopathy. 

Perineural spread is uncommon and is often detected incidentally. Commonly affected are the intra- or supraorbital branches of the trigeminal nerve, as seen in our patient, or the cervical and lumbosacral spinal nerves [7]. On CT, orbital pseudotumors associated with IgG4-RD demonstrate soft-tissue attenuation with homogenous enhancement. MRI reveals low signal intensity on T2-weighted images and hyperintensity on diffusion-weighted images, reflecting cellularity and fibrosis [13]. Therefore, systematic radiological evaluation along the course of cranial nerves is crucial for diagnosis. 

To our knowledge, this case represents the seventh report and fourth documented case of orbital IgG4-RD with perineural spread [13-15], excluding studies identifying periorbital nerve lesions retrospectively in patients with pre-existing IgG4-RD of other organs. In one, the first case of an IgG4-related inflammatory pseudotumor (IPT) of the trigeminal nerve was presented without involvement of the preferential head and neck sites [16]. Retrospective analyses emphasize lateral rectus enlargement as a diagnostic clue, with trigeminal nerve involvement being a significant feature [17-19]. This case, along with prior reports, underscores the need to recognize the patterns of disease spread in IgG4-RD to facilitate timely diagnosis and treatment. 

Differential diagnoses 

IgG4-RD was distinguished from other conditions based on specific clinical and imaging findings. Despite its fibroinflammatory nature, IgG4-RD does not present with fever [2], and the presence of fever typically indicates an alternative diagnosis [5]. In this case, our patient's afebrile status and negative ANA test steered us away from sarcoidosis and lymphoma as potential diagnoses. Additionally, granulomatosis with polyangiitis was considered less likely, given the absence of ANCA. Lastly, unlike thyroid ophthalmopathy, which spares tendons, IgG4-RD orbital myositis involves both muscle bellies and tendons [16]. These distinctions allowed for a definitive diagnosis of IgG4-RD for our patient. 

## Conclusions

IgG4-related disease (IgG4-RD) is increasingly recognized as the underlying etiology in diverse clinical presentations, with orbital and neurological involvement emerging as important manifestations. To the best of our knowledge, this case represents the fourth reported instance in the literature highlighting the perineural spread of orbital IgG4-RD. This phenomenon can become more readily identifiable by radiologists as awareness of its characteristic imaging features grows. 

In this patient, a stepwise approach to medical management was undertaken, culminating in successful targeting of the underlying inflammatory process despite treatment-related adverse events. This emphasizes the critical need for individualized therapeutic strategies to achieve optimal outcomes in patients with this complex, multifaceted disease. We aim to use this case to underscore the importance of increased recognition of IgG4-RD to facilitate earlier diagnosis and timely, targeted treatment. 
